# Development and Pilot Implementation of Neotree, a Digital Quality Improvement Tool Designed to Improve Newborn Care and Survival in 3 Hospitals in Malawi and Zimbabwe: Cost Analysis Study

**DOI:** 10.2196/50467

**Published:** 2023-12-22

**Authors:** Hassan Haghparast-Bidgoli, Tim Hull-Bailey, Deliwe Nkhoma, Tarisai Chiyaka, Emma Wilson, Felicity Fitzgerald, Gwendoline Chimhini, Nushrat Khan, Hannah Gannon, Rekha Batura, Mario Cortina-Borja, Leyla Larsson, Msandeni Chiume, Yali Sassoon, Simbarashe Chimhuya, Michelle Heys

**Affiliations:** 1 Institute for Global Health, University College London, London, United Kingdom; 2 Population, Policy and Practice Research and Teaching Department, Great Ormond Street Institute of Child Health, University College London, London, United Kingdom; 3 Kamuzu Central Hospital, Lilongwe, Malawi; 4 Centre for Sexual Health and HIV/AIDS Research, University of Zimbabwe, Harare, Zimbabwe; 5 Biomedical Research and Training Institute, Harare, Zimbabwe; 6 Department of Infectious Disease, Imperial College London, London, United Kingdom; 7 Department of Child Adolescent and Women’s Health, University of Zimbabwe, Harare, Zimbabwe; 8 Snowplow Analytics, London, United Kingdom; 9 Neonatal Unit, Sally Mugabe Central Hospital, Harare, Zimbabwe

**Keywords:** mHealth, clinical decision support, quality improvement tool, costs, cost, economic, economics, decision support, costing, expenditure, child, children, pediatric, pediatrics, paediatric, paediatrics, preterm, premature, baby, babies, newborn, newborns, maternal, neonatal, mobile health

## Abstract

**Background:**

Two-thirds of the 2.4 million newborn deaths that occurred in 2020 within the first 28 days of life might have been avoided by implementing existing low-cost evidence-based interventions for all sick and small newborns. An open-source digital quality improvement tool (Neotree) combining data capture with education and clinical decision support is a promising solution for this implementation gap.

**Objective:**

We present results from a cost analysis of a pilot implementation of Neotree in 3 hospitals in Malawi and Zimbabwe.

**Methods:**

We combined activity-based costing and expenditure approaches to estimate the development and implementation cost of a Neotree pilot in 1 hospital in Malawi, Kamuzu Central Hospital (KCH), and 2 hospitals in Zimbabwe, Sally Mugabe Central Hospital (SMCH) and Chinhoyi Provincial Hospital (CPH). We estimated the costs from a provider perspective over 12 months. Data were collected through expenditure reports, monthly staff time-use surveys, and project staff interviews. Sensitivity and scenario analyses were conducted to assess the impact of uncertainties on the results or estimate potential costs at scale. A pilot time-motion survey was conducted at KCH and a comparable hospital where Neotree was not implemented.

**Results:**

Total cost of pilot implementation of Neotree at KCH, SMCH, and CPH was US $37,748, US $52,331, and US $41,764, respectively. Average monthly cost per admitted child was US $15, US $15, and US $58, respectively. Staff costs were the main cost component (average 73% of total costs, ranging from 63% to 79%). The results from the sensitivity analysis showed that uncertainty around the number of admissions had a significant impact on the costs in all hospitals. In Malawi, replacing monthly web hosting with a server also had a significant impact on the costs. Under routine (nonresearch) conditions and at scale, total costs are estimated to fall substantially, up to 76%, reducing cost per admitted child to as low as US $5 in KCH, US $4 in SMCH, and US $14 in CPH. Median time to admit a baby was 27 (IQR 20-40) minutes using Neotree (n=250) compared to 26 (IQR 21-30) minutes using paper-based systems (n=34), and the median time to discharge a baby was 9 (IQR 7-13) minutes for Neotree (n=246) compared to 3 (IQR 2-4) minutes for paper-based systems (n=50).

**Conclusion:**

Neotree is a time- and cost-efficient tool, comparable with the results from limited similar mHealth decision-support tools in low- and middle-income countries. Implementation costs of Neotree varied substantially between the hospitals, mainly due to hospital size. The implementation costs could be substantially reduced at scale due to economies of scale because of integration to the health systems and reductions in cost items such as staff and overhead. More studies assessing the impact and cost-effectiveness of large-scale mHealth decision-support tools are needed.

## Introduction

In 2020, around 2.4 million neonatal deaths occurred globally, with most of the deaths (75%) occurring during the first week of life [1]. Low coverage and poor quality of care are among the main factors contributing to the high burden of neonatal mortality in low- and middle-income countries (LMICs) [2-5], in particular in sub-Saharan Africa and South Asia, where the highest rates of neonatal mortality occur [1]. Shortages of trained health care professionals and inadequate access and adherence to clinical guidelines are the main barriers to high-quality newborn care in health care facilities in LMICs [6-9]. Most neonatal deaths can be prevented through implementing low-cost evidence-based interventions [3,10].

Computerized or electronic clinical decision-support systems (CDSSs) have shown to improve adherence to clinical guidelines and health outcomes [11], though evidence mainly comes from high-income settings. CDSSs are any type of electronic system designed to directly assist in clinical decision-making, utilizing patient-specific information to produce individualized assessments or suggestions that are subsequently presented to health care professionals for their deliberation [12]. Over the past decade, widespread adoption of smartphones and tablets has enabled clinical decision-support tools to be accessible to health care providers on mobile devices directly at the point of care [13]. This is especially significant in LMICs and among their remote and underserved populations, where resources are limited.

Despite the growing adoption of mobile health (mHealth) decision-support tools in LMICs, there is a lack of comprehensive evidence regarding their effectiveness. The limited existing evidence regarding use of these tools in LMICs suggests their potential to improve adherence to clinical guidelines and both the quality of maternal care and childcare at primary health care (PHC) centers [14-16] and hospitals [7]; these tools might also improve clinical outcomes [16]. There is, however, limited evidence on costs and cost-effectiveness of mHealth decision-support tools in LMICs. This evidence gap limits assessments of budget impact and value for money, deterring large-scale programmatic implementation and adoption. There is, however, some limited economic evidence on computerized CDSSs or mHealth decision-support tools addressing improvements in maternal care [4,17-20]. All these were implemented at a PHC level, that is, communities [17-19] and health centers [4,20], rather than in hospitals. Findings from these studies show that the costs vary by intervention type, and that these tools can be cost-effective, though these results are based on small-scale and short-term implementation of the interventions.

This paper presents the costs of a pilot implementation of Neotree, an mHealth clinical decision-support app [21]. The Neotree system is an Android-based, open-source, and fully integrated digital health intervention that enables immediate data capture by health care professionals (primarily nurses) at the bedside while simultaneously providing evidence-based clinical decision support and newborn care education [21,22]. The app operates on low-cost Android tablets or mobile phones at the hospital bedside and is used by health care professionals to support their care and treatment of small and sick newborns in 3 hospitals in Malawi and Zimbabwe. It aims to increase the performance of the health care professionals, and as a result enhance quality of care, by creating a platform to improve supervision, support, and motivation. Early pilot data from Zomba Central Hospital in Malawi demonstrated high usability, acceptability, and feasibility [23], with potential for electronic audits and feedback to drive quality improvement (eg, targets for hypothermia on admission) [24]. Subsequent pilot implementation evaluation in Malawi and Zimbabwe has shown similar high usability, acceptability, and feasibility, with both perceived and observed improvements in quality of care [24,25]. Neotree has been in use since implementation in November 2018 to support the care of over 30,000 babies by more than 1000 health care providers, with ongoing implementation evaluation [21,26]. Neotree has been designed as a holistic clinical decision-support tool to address the leading causes of neonatal mortality and morbidity. Clinical decision support is based on the best available evidence, clinical heuristics, and expert consensus [27] and currently includes the following pathways: resuscitation, thermoregulation, convulsions, low birth weight, prematurity, hypoglycemia, HIV, respiratory distress, neonatal encephalopathy, sepsis, syphilis, jaundice and congenital abnormalities, stabilization, and transfer. Neotree is currently being adopted for PHC settings in Malawi; in Zimbabwe, it is being integrated into the district and national health information systems.

This study aims to calculate the costs of Neotree in its first year of implementation, to estimate future costs at scale, and to estimate the time taken to deliver clinical care on admission and discharge when using Neotree compared to standard-of-care paper-based systems.

## Methods

### Study Design and Setting

Details on the wider development and pilot implementation of Neotree are presented elsewhere [21,26]. Below we explain the Neotree pilot implementation in 3 hospitals in Malawi and Zimbabwe: Kamuzu Central Hospital (KCH) in Malawi and Sally Mugabe Central Hospital (SMCH) and Chinhoyi Provincial Hospital (CPH) in Zimbabwe.

Neotree was implemented in SMCH in November 2018 as part of a quality improvement project [25] when neonatal mortality rates were 27 per 1000 births [28]. On average, 12,000 babies are delivered at SMCH annually, which is the largest of 3 tertiary neonatal units in Zimbabwe. In 2019, 2985 babies were admitted to the neonatal intensive care unit for whom a matched outcome was recorded, with a case fatality rate (CFR) of around 177 deaths per 1000 admissions (unpublished data).

Neotree was subsequently implemented in KCH as a pilot study in April 2019, when neonatal mortality rates in Malawi were reported at 20.2 per 1000 births [28]. KCH is 1 of 4 central hospitals in Malawi. In 2019, approximately 3000 babies were delivered at KCH; 2732 babies were admitted to the neonatal unit, where the CFR was around 204 per 1000 admitted babies [29].

In October 2019, a 3-year mixed methods implementation evaluation commenced. In December 2020, Neotree was implemented at CPH, where an estimated 4500 babies are delivered annually with a CFR of 180 per 1000 babies admitted to the neonatal unit. In 2021, around 700 babies were admitted to the neonatal unit.

Neotree implementation in Malawi and Zimbabwe was supported by two nongovernmental organizations (NGOs). In each site, a project manager and 2 incentivized or salaried Neotree ambassadors were employed by the NGOs. The project managers’ role included both implementation and research (in their job plan, their time was equally split for these two roles). Neotree ambassadors were nursing staff who were paid a monthly salary supplement (100,000 Malawian kwacha [US $133], in Malawi) or full salary (US $1550, in Zimbabwe) to provide technical troubleshooting and implementation support. KCH (Malawi) had 2 Neotree ambassadors and SMCH and CPH (Zimbabwe) had 1 ambassador each but were supported by 5 incentivized nursing staff during weekends.

### Costing Approach

We used a combination of activity-based costing [30] and expenditure (top-down) approaches [31] to estimate the cost of developing a pilot implementation of Neotree at newborn care units in KCH, SMCH, and CPH. We estimated the costs from a provider perspective, including the cost of setting up and implementing Neotree at the hospitals (programmatic costs) and the cost to the hospital as a result of implementing Neotree. Costs to the hospitals were measured as opportunity (indirect) costs of the hospital staff involvement in the implementation of Neotree.

In addition, to explore the impact of Neotree on time spent on procedures and activities in the delivery of newborn care, a pilot time-motion survey was conducted at KCH and a comparable hospital in Malawi (Bwaila District Hospital) where Neotree was not implemented. Since at the time of data collection in 2020 and 2021 Neotree was used for admission and discharge at the newborn care units, we recorded only admission and discharge time for a number of admissions and discharges in each hospital (34 admissions and 50 discharges at Bwaila hospital, 250 admissions and 264 discharges at KCH). A trained nurse recorded start and end time for each procedure at Bwaila District Hospital in November 2021 and Neotree data were extracted from the Neotree app for the same period. The Wilcoxon-Mann-Whitney test was used to compare differences in admission and discharge times between the 2 hospitals.

**Figure 1.**
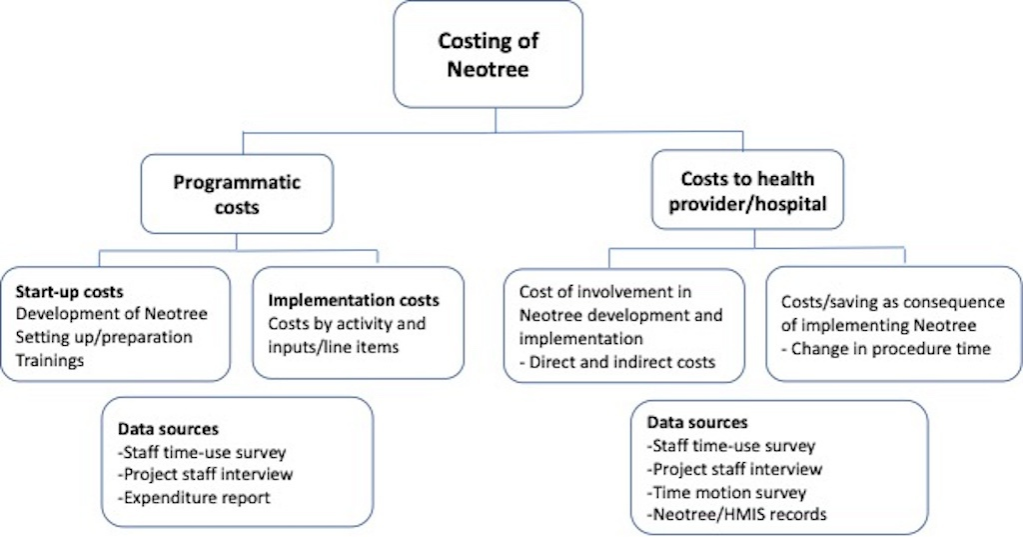
The conceptual framework for costing of Neotree. HMIS: Health Management Information System.

Figure 1 illustrates the conceptual framework used for the costing of Neotree. The initial time horizon for costing was 12 months (calendar year 2020 for KCH and SMCH, and 2021 for CPH). Programmatic cost data were collected through expenditure reports, monthly staff time-use surveys, and interviews with the project staff. Hospital-related costs were collected through staff time-use surveys, interviews with the project staff, and time-motion surveys. We estimated the economic cost of implementing Neotree, which included both financial costs (extracted from the expenditure reports) and opportunity costs of involving hospital staff in running Neotree.

An Excel-based costing tool was adapted from previous studies [32] to collect and analyze the cost data. The tool categorizes costs based on inputs or line items and activities. Main inputs or line items were staff, capital, material, web hosting, transportation, and overhead costs. Seven main activities identified through consultation with the implementation team were grouped into development and routine activities. Table 1 presents full descriptions of activities and line items/inputs. We excluded all research-related activities and costs, such as research meetings, as well as process and impact evaluations.

**Table 1.** Description of activities and inputs/line items.

Costs		Descriptions
**Costs by line item**		
	Staff	Value of staff time contributed in development and running of Neotree; staff costs mainly include incentives and salary paid to Neotree ambassadors (n=4), incentives paid to nurses for temporary weekend cover (in Zimbabwe), and the salary of the project manager at each site (n=2)
	Capital	Costs of tablets and other equipment used in running Neotree
	Materials—running Neotree	Costs of materials related to running Neotree
	Materials—other	Costs of materials such as office supplies, refreshments related to meetings, and training
	Transportation	Includes items such as costs of fuel and maintenance
	Travel	Includes cost items such as per diem and other allowances
	Overhead	Include cost items such as running costs and rent, utilities, communications, recruitment costs, and other overhead or joint costs
	Web hosting	Cost of monthly web hosting (in Malawi only)^a^
**Costs by activity**		
	Neotree data pipeline	Data pipeline testing and quality assurance
	Data backup	The process of manually backing up data, which entails exporting data from each tablet to AWS^b^, then moving the Excel files from the tablets to a laptop, and then uploading them to the server
	Roll out and setting up	Includes training and setting up activities
	Admission and discharge or death data entry	The amount of time HCPs^c^ take to do data entry during patient admission and outcomes; outcomes include being discharged alive, discharged on request, discharged on palliative care, death at less than or more than 24 hours of life, being transferred out, and other outcomes
	MM^d^ dashboard preparation	Includes monthly data analysis, developing visuals from the monthly admission and outcome data, and preparing a PowerPoint presentation for the HCPs to use during the MM monthly meeting
	Neotree support	Includes providing support and troubleshooting with the tablets, data dashboards, and printers, as well as updating or editing content in the app
	Data quality checks/data audits	Includes running some SQL queries on the data to check anomalies, especially the patient unique identifier, and checking the physical files to verify any missing outcomes, especially death outcomes
	Joint activities/administration	Staff time spent on administration or joint activities, such as joint monthly meetings
	Monthly software maintenance	Cost of monthly software maintenance by a software developer

a Neotree pilot started at Kamuzu Central Hospital in 2019. Fom the beginning, data were stored on a cloud server (Amazon Web Services) with monthly charges. However, in 2 hospitals in Zimbabwe, we used a physical server to store data, which was less costly. These data storage solutions were developed to adhere to data regulations and preferences within each country.

b AWS: Amazon Web Services.

c HCP: health care professional.

d MM: morbidity and mortality.

The activities were defined as cost centers, and all costs were allocated to them. Staff costs were allocated to the activities using time-use data collected from all staff involved in running Neotree. The same allocation rule was used to allocate joint costs, such as web hosting and overhead costs of the activities.

In Zimbabwe, all costs were measured in US dollars, and in Malawi, all costs were converted to US dollars using the 2020 exchange rate of US $ 1=749.53 kwacha [33]. Capital costs were annualized using the expected life of each item and a discount rate of 5% [34].

We present the total economic and financial costs of developing and running Neotree, average monthly costs, and average monthly costs per admitted newborn at the 3 pilot hospitals.

### Sensitivity and Scenario Analyses

We conducted a number of 1-way sensitivity analyses assessing the impact of uncertain variables or parameters on the results. These parameters are the discount rates for capital (3%, 5%, and 10%), exchange rate (±25%, only for Malawi), replacing web hosting with a server (in KCH, Malawi), uncertainty around implementation costs (±25%), and uncertainty around total number of admissions (min-max). We also analyzed a number of scenarios (described below and in Table 2) that reflect potential costs of Neotree at scale.

**Table 2.** Scenario analysis summary.

	Base case	Scenario 1 (routine costs)	Scenario 2	Scenario 3
Development activities	✓			
Neotree ambassadors	✓			
Project managers	NGO^a^ salaries and 12% overhead	NGO salaries and 12% overhead	Hospital/MoH^b^ salaries and 5% overhead	Hospital/MoH salaries and 5% overhead
Project management and server costs shared across multiple sites				✓

a NGO: nongovernmental organization.

b MoH: Ministry of Health.

#### Scenario 1 (Routine Conditions)

In costing the pilot implementation of Neotree, we have included monthly allowances or salary paid to the Neotree ambassadors (US $133 in KCH to US $1550 in SMCH and CPH) to provide technical troubleshooting and implementation support. Under routine conditions, or at Neotree’s roll out, these routine activities will be conducted by staff without receiving an allowance. In addition, we considered that no development activities would be conducted under routine conditions, and as such these costs were removed under this scenario.

#### Scenario 2

The project managers in both settings were employed by the 2 NGOs, that is, they were paid at the NGO pay scale. In addition, a portion of the NGOs’ overhead costs (12% in both settings, regardless of number of hospitals supported) were included in the implementation costs. At scale, it is likely that Neotree would be run by public hospitals, and therefore staff would be paid at the ministry of health (MoH) pay scale and overhead costs would be substantially lower. Therefore, in this scenario, we replaced the salary of the project managers with the salary of hospital staff with equivalent skills, and we assumed that the overhead costs would be reduced to 5%. Under this scenario, we used the estimates from scenario 1 (routine costs) and reduced overhead costs.

#### Scenario 3 (Potential Costs at Scale)

At scale, costs such as project management or support and servers can be shared across a number of hospitals. In Zimbabwe, the server was already used for both hospitals and the support costs were the same. We assumed these costs could be shared between 4 hospitals at scale.

### Ethical Considerations

Ethics approvals for the Neotree pilot implementation and associated feasibility study data collection were obtained from the Malawi College of Medicine Research and Ethics Committee (P.01/20/2909; P.02/19/2613), University College London (17123/001, 6681/001, 5019/004), the Medical Research Council of Zimbabwe (MRCZ/A/2570), the Biomedical Research and Training Institute and Joint Research Ethics Committee for the University of Zimbabwe institutional review boards (AP155/2020; JREC/327/19), and the Sally Mugabe Hospital Ethics Committee (071119/64; 250418/48).

## Results

### Cost Data

Table 3 presents the total economic costs of developing and implementing a pilot of Neotree at 3 hospitals. Tables S1 and S2 in Multimedia Appendix 1 present the financial costs of implementing Neotree. The total economic cost of pilot implementation in the 3 hospitals ranged from US $37,748 in KCH to US $52,331 in SMCH (Table 3). Considering average monthly admissions at newborn care units in the hospitals, the average monthly costs per admitted newborn ranged from US $15 in KCH and SMCH to US $58 in CPH, mainly reflecting the size of the hospitals.

**Table 3.** Total economic costs of the Neotree pilot implementation in Kamuzu Central Hospital (KCH), Sally Mugabe Central Hospital (SMCH), and Chinhoyi Provincial Hospital (CPH). Total cost represents the cost of all development and routine activities conducted in 12 months. Costs are estimated in 2020 US $ for KCH and SMCH and in 2021 US $ for CPH.

	KCH	SMCH	CPH
Total cost, US $	37,748	52,331	41,764
Average monthly costs, US $	3146	4361	3480
Average monthly admissions, n	210	298	60
Average monthly costs per admission, US $	15	15	58

Staff costs constituted on average 73% of total costs, ranging from 63% in KCH to 79% in CPH. The category “overhead costs” was the second highest input, with around 13% of total costs. However, in KCH, web hosting costs constituted around 17% of total costs (Table 4 and and Table S1 in Multimedia Appendix 1). In terms of activities, routine activities comprised around 72% of total costs, where support and data entry activities were on average the main cost drivers among these routine activities. However, the proportion of these activities vs the total routine costs varied substantially in each hospital (Table 5 and Table S2 in Multimedia Appendix 1).

**Table 4.** Total economic costs of Neotree pilot implementation in Kamuzu Central Hospital (KCH), Sally Mugabe Central Hospital (SMCH), and Chinhoyi Provincial Hospital (CPH) by line item/input. Total cost represents the cost of all development and routine activities conducted in 12 months. Costs are estimated in 2020 US $ for KCH and SMCH and in 2021 US $ for CPH.

Line item/input	Costs at KCH (total=US $37,748), US $ (%)	Costs at SMCH (total=US $52,331), US $ (%)	Costs at CPH (total=US $41,764), US $ (%)
Staff	23,696 (63)	40,524 (77)	32,995 (79)
Capital	941 (2)	1602 (3)	1408 (3)
Materials	1331 (4)	3600 (7)	2100 (5)
Transport/travel	161 (0)	192 (0)	153 (0)
Web hosting	6600 (17)	N/A^a^	N/A
Overhead	5019 (13)	6413 (12)	5109 (12)

a N/A: not applicable. Web-hosting costs were only incurred in KCH; SMCH and CPH used a physical server.

**Table 5.** Total economic costs of Neotree pilot implementation in Kamuzu Central Hospital (KCH), Sally Mugabe Central Hospital (SMCH), and Chinhoyi Provincial Hospital (CPH) by main activity. Total cost represents costs of all development and routine activities conducted in 12 months. Costs estimated in 2020 US $ for KCH and SMCH and in 2021 US $ for CPH.

Activities		Costs at KCH (total =US $37,748), US $ (%)	Costs at SMCH (total =US $52,331), US $ (%)	Costs at CPH (total =US $41,764), US $ (%)
**Development activities**				
	Neotree data pipeline	5003 (13)	0 (0)	4121 (10)
	Data backup	1714 (5)	10,524 (20)	6033 (14)
	Rollout	2460 (7)	4245 (8)	3456 (8)
**Routine activities**				
	Data entry	2355 (6)	13,014 (25)	12,624 (30)
	Morbidity and mortality dashboard preparation	1309 (3)	2654 (5)	7569 (18)
	Neotree support	14,828 (39)	10,909 (21)	2373 (6)
	Data quality checks/audits	3073 (8)	6810 (13)	4854 (12)
	Maintenance	7007 (19)	4175 (8)	734 (2)

### Sensitivity and Scenario Analyses

The results from a 1-way sensitivity analysis showed that uncertainty around the number of admissions had a significant impact on costs in all hospitals, ranging from −38% to +239%. The varying exchange rate in Malawi also imposed a significant cost, ranging from −19% to +31% (Table S3 in Multimedia Appendix 1). Similarly, replacing monthly web hosting with an in-country physical server reduced the total cost by 16% in Malawi.

The results from the scenarios showed that, on average, compared to the base case scenario, the total costs would be reduced by 56%, 66%, and 70% under scenarios 1, 2, and 3, respectively (Table 6). Under scenario 1 (routine conditions), total costs in the hospitals would be reduced on average by 56%, ranging from 45% in KCH to 67% in CPH. For example, the cost per admitted child would be reduced from US $15 to US $8 in KCH, from US $15 to US $6 in SMCH, and from US $58 to US $19 in CPH.

**Table 6.** The results from scenario analyses of Neotree pilot implementation in Kamuzu Central Hospital (KCH), Sally Mugabe Central Hospital (SMCH), and Chinhoyi Provincial Hospital (CPH).

Hospitals and scenarios		Total costs, US $	Average monthly costs, US $	Total costs per admission, US $
**KCH**				
	Base case scenario	37,748	3146	15
	Scenario 1	20,745	1729	8
	Scenario 2	16,459	1372	7
	Scenario 3	13,711	1143	5
**SMCH**				
	Base case scenario	52,331	4361	15
	Scenario 1	22,801	1900	6
	Scenario 2	17,602	1467	5
	Scenario 3	14,612	1218	4
**CPH**				
	Base case scenario	41,764	3480	58
	Scenario 1	13,730	1144	19
	Scenario 2	10,746	895	15
	Scenario 3	10,150	846	14

Implementing Neotree with hospital or MoH staff (scenario 2), would further reduce costs by 22% on average (ranging from 21% in KCH to 23% in SMCH) in the hospitals compared with scenario 1. Similarly, under the potential scaled-up scenario (scenario 3), the total costs would be further reduced by 13% (ranging from 6% in CPH to 17% in KCH and SMCH) compared to scenario 2 (Table 6). Under this scenario, cost per admission would be $5, $4, and $14 in KCH, SMCH, and CPH, respectively.

### Time-Motion Survey Results

The results from our time-motion study on these 2 procedures using Neotree (KCH) and a paper-based system (Bwaila District Hospital) showed that the median time to admit a baby was 27 (IQR 20-40) minutes (n=250) using Neotree and 26 (IQR 21-30) minutes (n=34) using the paper-based system, while the mean time to discharge a baby was 9 (IQR 7-13) minutes (n=246) using Neotree and 3 (IQR 2-4) minutes (n=50) using the paper-based system (Figure S1 in Multimedia Appendix 1).

There was no statistically significant difference between admission times between these two systems (*P=*.55) but there was a significant difference in discharge times (*P=*.001). Opportunity costs of the additional time spent on discharging a newborn using Neotree were around US $0.71, ranging from US $0.44 in KCH to US $1 in SMCH.

## Discussion

### Principal Findings

Our study estimates the costs of a pilot implementation of Neotree, an mHealth clinical decision-support app, in 3 hospitals in Malawi and Zimbabwe. It contributes to the limited cost and cost-effectiveness data for mHealth decision-support tools in LMICs. To our knowledge, this is the first study reporting cost data for a digital newborn care intervention in LMICs. Total cost of pilot implementation of Neotree ranged from US $37,748 to US $52,331. Taking into account average monthly newborn admissions in the hospitals, the average monthly cost per admitted child ranged from US $15 to US $58, which mainly reflects the size of the newborn care units. However, under routine conditions and at scale, these costs will be reduced substantially, up to 76%, reducing cost per admitted child to as low as US $5 in KCH (Malawi), US $4 in SMCH, and US $14 in CPH (the latter two both in Zimbabwe).

### Findings Compared With Other mHealth Decision-Support Tools

Comparing costs of Neotree with other mHealth decision-support tools is challenging because of differences in type of intervention (eg, computerized CDSS or mHealth app), implementation site (eg, hospital, PHC center, or community), scale, and costing approach. To our knowledge, there are no published cost data on hospital-based implementations of mHealth decision-support tools in LMICs for any clinical cohort. However, the results from Neotree are comparable with 2 computer-assisted CDSSs piloted in PHC centers in Ghana [35] and Tanzania [36]. These 2 studies were part of the QUALMAT (Quality of prenatal and maternal care: bridging the know-do gap) project piloting computer-assisted CDSSs in PHC centers in a number of countries in sub-Saharan Africa. In Ghana, the tool was piloted in antenatal clinics and the labor wards of 6 PHC centers and implemented by trained nurses. During a 1-year pilot implementation, 22 nurses were trained on a CDSS, and 5595 antenatal consultations (44% of total consultations) and 872 labor patients (60% of total patients) were managed using the CDSS. The economic cost of the intervention was 2012 US $17,129 (or 2020 US $13,935), which included an approximately 2.5-year preintervention and a 1-year intervention implementation. Costs per antenatal consultation and labor care were 2012 US $3 (or 2020 US $3) and 2012 US $20 (or 2020 US $16), respectively [35]. In Tanzania, the tool was piloted for antenatal care in 6 PHC centers (5 public and 1 private). The economic cost of installing and piloting the intervention was 2013 US $127,506 (or 2020 US $121,914), including an approximately 2.5-year preintervention and a 1-year intervention implementation. During the 1-year implementation, 1665 antenatal contacts (70% of total contacts) and 754 childbirths (85% of total childbirths) were registered in the CDSS. Cost per total contact was 2013 US $53 (2020 US $50) [36].

Follow up cost-effectiveness analyses of these 2 interventions have shown that they were potentially cost-effective. In Ghana, the incremental cost-effectiveness ratio (ICER) of a computer-assisted CDSS compared to a paper-based system was estimated at 2012 US $1142 per pregnancy complication detected. Considering only additional costs implementing computer-assisted CDSSs, the cost per pregnancy complication detected was US $285 [20]. In Tanzania, ICERs were 2013 US $2469 and US $338 per 1% change in process quality for antenatal and childbirth care, respectively [4].

It should be taken into account that the cost evidence for Neotree and QUALMAT studies are from 1-year pilot studies and do not capture long-term economic and noneconomic impacts of the interventions. Economic evidence from long- and short-term implementation of CDSSs in high-income countries has shown promising results in reduction of health care expenditures, such as through reducing unnecessary laboratory testing and antibiotic prescriptions. However, the quality of these studies has been variable [37,38].

In terms of cost profile, staff costs constituted most of Neotree’s implementation costs (on average 71%), followed by overhead costs (around 13%). This cost profile is similar to the QUALMAT study in Ghana [35], with staff constituting 39% and overhead 23% of total costs, followed by training (16%) and equipment (10%). In Tanzania, training costs constitute 48% of total costs, followed by staff costs with 22% [36].

### Implications

The substantial differences in the average monthly cost of Neotree per admitted child, which varied from US $15 to US $58, mainly reflects the size of the newborn care units in the hospitals and thus the number of admitted babies. CPH, as a provincial hospital, has the smallest unit and the lowest number of admissions compared with KCH and SMCH (both are central, ie, tertiary hospitals). As we have shown in our scenario analyses, these unit costs can be significantly reduced when implemented as part of health system strengthening and at scale (ranging from US $4 to US $14). Implementation of an intervention as part of a health system has a distinct cost advantage. Implementation cost at scale can be substantially lower due to potential economies of scale on cost items such as maintenance and data hosting costs (in the case of KCH in Malawi). Some coordination and overhead costs will be reduced if Neotree is run directly by hospitals supported by the existing infrastructure without coordinating with an external agency, as was done in the Neotree pilot implementation. It should be acknowledged that the estimated costs might be generalizable to similar hospitals in Malawi and Zimbabwe but not necessarily to other facility types or settings other than these 2 countries.

In earlier studies, we noted a concern from HCPs that admitting and discharging a baby using Neotree might take an HCP (typically a nurse) too much time, thus distracting them from the care of other babies in the unit [23]. Human resources and capacity have been reported as key determinants of quality of newborn care [9]. Our pilot time-motion data suggest this has not been the case—at least in Malawi. Arguably, babies admitted to Bwaila District Hospital should be of lower clinical acuity (as it is a smaller hospital); however, our data show no statistically significant difference in time taken to admit a baby. Nevertheless, continual monitoring is required as the addition of more comprehensive clinical decision-support features may increase the time taken to complete clinical procedures (eg, to admit a baby).

### Limitations

Our study has a number of potential limitations. We estimated costs from a provider perspective, including costs of developing and implementing Neotree and the opportunity cost of hospital staff contribution. However, we were not able to capture the full development cost of Neotree, as the first prototype for Neotree was developed in 2013, and it was further developed in 2016 and 2019. However, we have tried to reflect this in our sensitivity analysis. In addition, we were not able to measure the full impact of Neotree on the hospital or health system due to the short time horizon. For example, we did not measure the impact of Neotree on costs or savings due to changes in procedure times other than admission and discharge, nor did we measure savings due to reductions in the time needed for blood culture results (from 6 day to 3 days) or clinical auditing and quality review (from few days to few hours), nor the potential impact on tests and medication prescriptions. Lastly, due to the volatile nature of inflation in Zimbabwe, it was not possible to deflate the cost of CPH to 2020 to be comparable with KCH and SMCH.

### Conclusion

Our findings show that Neotree is a time-efficient and cost-efficient tool, comparable with the results from limited mHealth clinical decision-support tools in LMICs. The implementation cost of Neotree varied substantially between the hospitals, mainly affected by the size of the hospitals. Our analysis showed that Neotree implementation costs can be substantially reduced at scale due to potential economies of scale as a result of integration to the health system and reductions in cost items such as staff and overhead. More studies assessing the impact and cost-effectiveness of larger-scale mHealth decision-support tools such as Neotree are needed.

## Supplementary material

10.2196/50467 Multimedia Appendix 1 Supplementary tables and figure.

## Abbreviations

AWS: Amazon Web Services
CDSS: clinical decision-support system
CFR: case fatality rate
CPH: Chinhoyi Provincial Hospital
HCP: health care professional
ICER: incremental cost-effectiveness ratio
KCH: Kamuzu Central Hospital
LMIC: low- and middle-income country
MCH: Sally Mugabe Central Hospital
mHealth: mobile health
MM: morbidity and mortality
NGO: nongovernmental organization
PHC: primary health care
QUALMAT: Quality of prenatal and maternal care: bridging the know-do gap
