# Supplementary material for: Development and Pilot Implementation of Neotree, a Digital Quality Improvement Tool Designed to Improve Newborn Care and Survival in 3 Hospitals in Malawi and Zimbabwe: Cost Analysis Study
Source: JMIR Mhealth Uhealth. 2023 Dec 22;11:e50467. doi: 10.2196/50467 (PMC10766148; doi:10.2196/50467)
Supplement: Multimedia Appendix 1 [file mhealth-v11-e50467-s001.docx]

Table S1: Total financial costs of Neotree pilot implementation in Kamuzu Central Hospital (KCH), Sally Mugabe Central Hospital (SMCH) and Chinhoyi Provincial Hospital (CPH), by line item*

| Line item/input | KCH | % | SMCH | % | CPH | % |
| --- | --- | --- | --- | --- | --- | --- |
| Staff | 23,486 | 63% | 40,524 | 78% | 32,995 | 79% |
| Capital | 865 | 2% | 1,418 | 3% | 1,239 | 3% |
| Materials | 1,331 | 4% | 3,600 | 7% | 2,100 | 5% |
| Transport/travel | 161 | 0% | 191 | 0% | 152 | 0% |
| Hosting | 6,600 | 18% | - | 0% | - | 0% |
| Overhead | 5,019 | 13% | 6,384 | 12% | 5,083 | 12% |
| Total costs | **37,461** | 100% | **52,116** | 100% | **41,568** | 100% |

* Total costs is costs of all development and routine activities conducted in 12 months. Costs estimated in 2020 US$ for KCH and SMCH and in 2021 US$ for CPH.

Table S2: Total financial costs of Neotree pilot implementation in Kamuzu Central Hospital (KCH), Sally Mugabe Central Hospital (SMCH) and Chinhoyi Provincial Hospital (CPH), by main activity*

| Activity/Year | | KCH | SMCH | CPH |
| --- | --- | --- | --- | --- |
| *Development* | NeoTree data pipeline | 4,983 | - | 4,118 |
|  | Data back-up | 1,714 | 10,518 | 6,029 |
| *Roll out/set up* |  | 2,460 | 4,245 | 3,456 |
| *Routine* | Data Entry (Admission and Discharges/Deaths) | 2,355 | 12,946 | 12,532 |
|  | M&M dashboard preparation | 1,303 | 2,637 | 7,526 |
|  | NeoTree Support | 14,568 | 10,836 | 2,350 |
|  | Data Quality Checks/audit | 3,073 | 6,771 | 4,827 |
|  | Monthly maintenance | 7,007 | 4,164 | 730 |
| Total costs | | **37,461** | **52,116** | **41,568** |

* Total costs is costs of all development and routine activities conducted in 12 months. Costs estimated in 2020 US$ for KCH and SMCH and in 2021 US$ for CPH.

Table S3: Results from one-way sensitivity analysis of Neotree implementation in Kamuzu Central Hospital (KCH), Sally Mugabe Central Hospital (SMCH) and Chinhoyi Provincial Hospital (CPH), by main activity (US$)*.

| Hospitals | Parameters | Total costs | Average monthly costs | Total costs per admission |
| --- | --- | --- | --- | --- |
| KCH | **Base-case** | 37,748 | 3,146 | 15.00 |
|  | **Varying discount rate for capital costs** (Base-case: 5%) | | | |
|  | 3% | 37,717 | 3,143 | 14.99 |
|  | 10% | 37,826 | 3,152 | 15.03 |
|  | **Exchange rate +/-25%** | | | |
|  | +25% | 30,690 | 2,558 | 12.20 |
|  | -25% | 49,511 | 4,126 | 19.68 |
|  | **Implementation costs +/-25%** | | | |
|  | +25 | 47,185 | 3,932 | 18.75 |
|  | -25 | 28,311 | 2,359 | 11.25 |
|  | **Total number of admissions, min-max (base-case=210)** | | | |
|  | Min: 142 | N/A | N/A | 22.15 |
|  | Max: 287 | N/A | N/A | 10.96 |
|  | **Replacing hosting with server in Malawi** | 31,607 | 2,634 | 12.56 |
| SMCH | **Base-case** | 52,331 | 4,361 | 14.63 |
|  | **Varying discount rate for capital costs** (Base-case: 5%) | | | |
|  | 3% | 52,243 | 4,354 | 14.61 |
|  | 10% | 52,556 | 4,380 | 14.70 |
|  | **Implementation costs +/-25%** | | | |
|  | +25 | 65,413 | 5,451 | 18.29 |
|  | -25 | 39,248 | 3,271 | 10.97 |
|  | **Total number of admissions, min-max (base-case=298)** | | | |
|  | Min: 88 | N/A | N/A | 50 |
|  | Max: 477 | N/A | N/A | 9 |
| CPH | **Base-case** | 41,764 | 3,480 | 58 |
|  | **Varying discount rate for capital costs** (Base-case: 5%) | | | |
|  | 3% | 41,685 | 3,474 | 58 |
|  | 10% | 41,971 | 3,498 | 58 |
|  | **Implementation costs +/-25%** | | | |
|  | +25 | 52,206 | 4,350 | 72 |
|  | -25 | 31,323 | 2,610 | 43 |
|  | **Total number of admissions, min-max (base-case=60)** | | | |
|  | Min: 28 | N/A | N/A | 124 |
|  | Max: 82 | N/A | N/A | 42 |

* Total costs is costs of all development and routine activities conducted in 12 months. Costs estimated in 2020 US$ for KCH and SMCH and in 2021 US$ for CPH.

Figure S1: Results from the time motion survey
